# Motion of the Esophagus Due to Cardiac Motion

**DOI:** 10.1371/journal.pone.0089126

**Published:** 2014-02-28

**Authors:** Jacob Palmer, Jinzhong Yang, Tinsu Pan, Laurence E. Court

**Affiliations:** Department of Radiation Physics, Unit 94, The University of Texas MD Anderson Cancer Center, Houston, Texas, United States of America; Northwestern University Feinberg School of Medicine, United States of America

## Abstract

When imaging studies (e.g. CT) are used to quantify morphological changes in an anatomical structure, it is necessary to understand the extent and source of motion which can give imaging artifacts (e.g. blurring or local distortion). The objective of this study was to assess the magnitude of esophageal motion due to cardiac motion. We used retrospective electrocardiogram-gated contrast-enhanced computed tomography angiography images for this study. The anatomic region from the carina to the bottom of the heart was taken at deep-inspiration breath hold with the patients' arms raised above their shoulders, in a position similar to that used for radiation therapy. The esophagus was delineated on the diastolic phase of cardiac motion, and deformable registration was used to sequentially deform the images in nearest-neighbor phases among the 10 cardiac phases, starting from the diastolic phase. Using the 10 deformation fields generated from the deformable registration, the magnitude of the extreme displacements was then calculated for each voxel, and the mean and maximum displacement was calculated for each computed tomography slice for each patient. The average maximum esophageal displacement due to cardiac motion for all patients was 5.8 mm (standard deviation: 1.6 mm, maximum: 10.0 mm) in the transverse direction. For 21 of 26 patients, the largest esophageal motion was found in the inferior region of the heart; for the other patients, esophageal motion was approximately independent of superior-inferior position. The esophagus motion was larger at cardiac phases where the electrocardiogram R-wave occurs. In conclusion, the magnitude of esophageal motion near the heart due to cardiac motion is similar to that due to other sources of motion, including respiratory motion and intra-fraction motion. A larger cardiac motion will result into larger esophagus motion in a cardiac cycle.

## Introduction

Many groups have investigated the respiratory motion of radiation targets (i.e., tumors) and normal tissues in the thoracic cavity and the impact of this motion in diagnostic imaging and radiation therapy [1], [2], [3], [4], [5], [6]. Only a few studies have assessed the effect of cardiac motion in radiation therapy [1], [3], [7], and, to our knowledge, none have assessed the impact of cardiac motion on esophageal motion. The effects from cardiac and respiratory motions may not generally be separable as most therapy sessions are performed with free-breathing. Also, although respiratory motion can be minimized by either breathhold techniques or abdominal compression, any motion due to cardiac pulsing will remain. This may give a blurring of the dose in radiation therapy (where treatments are several minutes long); the effect in imaging studies will depend on the temporal resolution of the imaging study, but could include blurring or local geometric distortion. This may be particularly important for longitudinal studies that attempt to examine local morphological changes at the voxel level.

There are several reasons why it may be important to accurately image the esophagus – some examples in radiation therapy are given here. The esophagus can be an important dose-limiting structure in radiation therapy treatment planning, but its dose-response characteristics have not been well characterized. That is, although it is known that volumes of the esophagus receiving more than 40–50 Gy correlate significantly with acute esophagitis [8], the impact of the spatial distribution is not yet understood. Some studies have shown that circumferential metrics (i.e., the esophageal length receiving a certain full-circumference dose) are statistically significant [9], [10], but they have not been shown to be superior to dose-volume metrics. One likely reason for this is that the spatial extent (location) and severity of esophagitis is currently unknown, so easily determining the exact impact of the spatial dose distribution on the local tissue is not possible. Several imaging techniques could be used to do this on a voxel-by-voxel basis, thus allowing the calculation of a true voxel-by-voxel dose-response curve. One such technique uses computed tomography (CT) images to measure the esophageal swelling that occurs as a result of radiation damage [11]. Another possible technique is the use of positron emission tomography (PET) images. Although this second technique has yet to be used to assess esophageal toxicity, it has been successfully used to show a relationship between dose and standardized uptake values (SUV) for radiation-induced lung toxicity [12]. The effects of motion (attributed to respiration or some other source) on these techniques is complex, and will depend on details of the imaging technique used, but could include blurring (depending on temporal resolution) or local geometric distortions in the esophageal shape. These effects could be particularly relevant when trying to assess changes in the esophagus (CT or PET, etc.) on a voxel-by-voxel level). Thus, it is important to understand the effects of motion prior to using imaging (CT or PET) to understand the dose-response of the esophagus. Therefore, we initiated this study to assess the impact of cardiac motion on the esophagus as an important step toward understanding the different causes of motion uncertainty.

## Materials and Methods

### Patient data

A total of 26 patients who had received contrast-enhanced CT-based coronary angiography with retrospective electrocardiographic gating had been randomly selected in a previous study [7]. The use of these data for the current study was approved by the Institutional Review Board at The University of Texas MD Anderson Cancer Center (PA12-0340), including a specific waiver to obtain written consent from the patient for this retrospective data review study. The images were taken using a GE LightSpeed VCT 64-slice CT scanner (GE Healthcare, Waukesha, WI) and deep-inspiration breath hold with patients' arms raised above their shoulders, in a position similar to that used for radiation therapy. For each patient, 10 CT data sets were reconstructed over a cardiac cycle from the carina to the bottom of the heart. These images had 1.25-mm slice thickness and 0.35×0.35 mm^2^ pixel size. The images corresponding to the diastolic phases of cardiac motion were imported into a Pinnacle^3^ treatment planning system (Philips Medical Systems, Fitchburg, WI), and the esophagus was delineated on a single, consistent phase using standard delineation tools.

### Motion analysis

**Figure 1:**
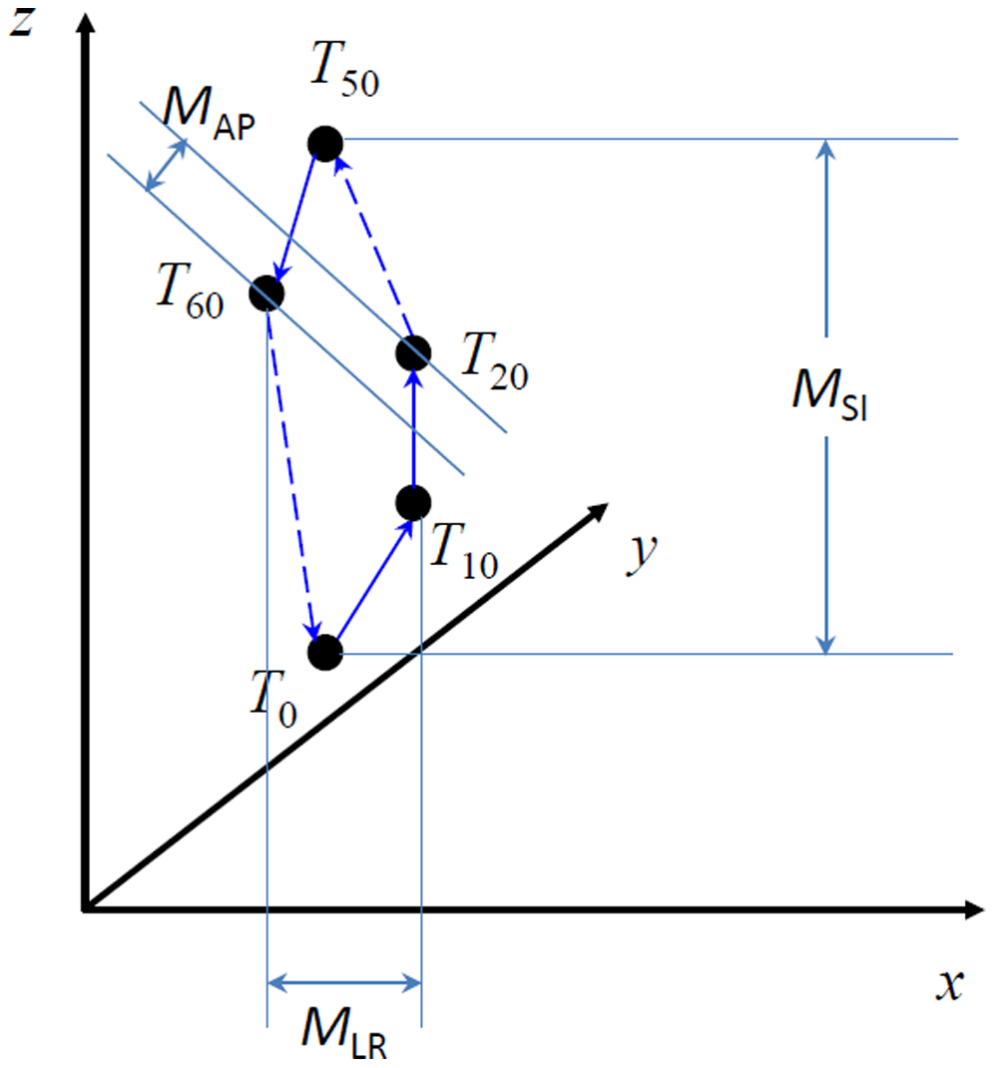
Illustration of voxel-level motion trajectory. The displacement vectors from 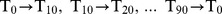 are concatenated together to form this motion trajectory. The motion magnitudes in three directions, 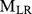, 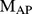, and 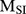, were computed by measuring the maximum range of the trajectory in all three directions: left-right (LR), anterior-posterior (AP), and superior-inferior (SI).



For each patient the image sets comprised 10 CT data sets (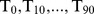) over a cardiac cycle. (The images are reconstructed from multiple cardiac cycles. The phases of the images are over a cardiac cycle from 0% to 90% like the ones in 4D-CT.) We used an in-house deformable image registration tool to perform the motion analysis based on the 10 phases of CT images. An accelerated Demons algorithm [13] was applied to sequentially register the 10 CT images and generate 10 displacement fields (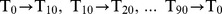). We validated the deformation visually by comparing the deformed images with the original image. The 10 displacement fields were concatenated to form the motion trajectory for each voxel in a cardiac cycle. The voxel-level motion trajectory is illustrated in Figure 1. The motion magnitudes in three directions—left-right (LR), anterior-posterior (AP), and superior-inferior (SI)—were computed for each voxel by measuring the maximum range of the trajectory in all three directions. The total motion magnitude for each voxel was computed as

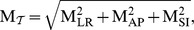
where 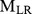, 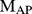, and 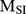, are the motion magnitudes in the LR, AP, and SI directions, respectively. The motion trajectory for voxels inside the esophagus contour constitutes the esophageal motion. For each patient, and for each CT slice, the mean and maximum motions at each direction (LR, AP, and SI) and the total motion magnitudes were calculated. Then, for each patient, the maximum values of these mean and maximum motions over all slices were calculated. Finally, the mean, standard deviation, and maximum range of these values were calculated for the overall patient population. Note, when calculating the motion in a slice, we noticed that the motion in SI direction was not reliable and difficult to validate its accuracy. Therefore, we reported the motion in the transverse direction without taking into account the SI motion. In addition, the esophagus motion of each cardiac phase was analyzed, which might be beneficial for determining the optimal imaging temporal resolution or acquisition phase in a future study. For each patient, the maximum motion of esophagus was determined from the 10 displacement fields for each phase, and the averaged maximum motion was calculated from the overall patient population for comparison among different phases.

## Results

**Figure 2:**
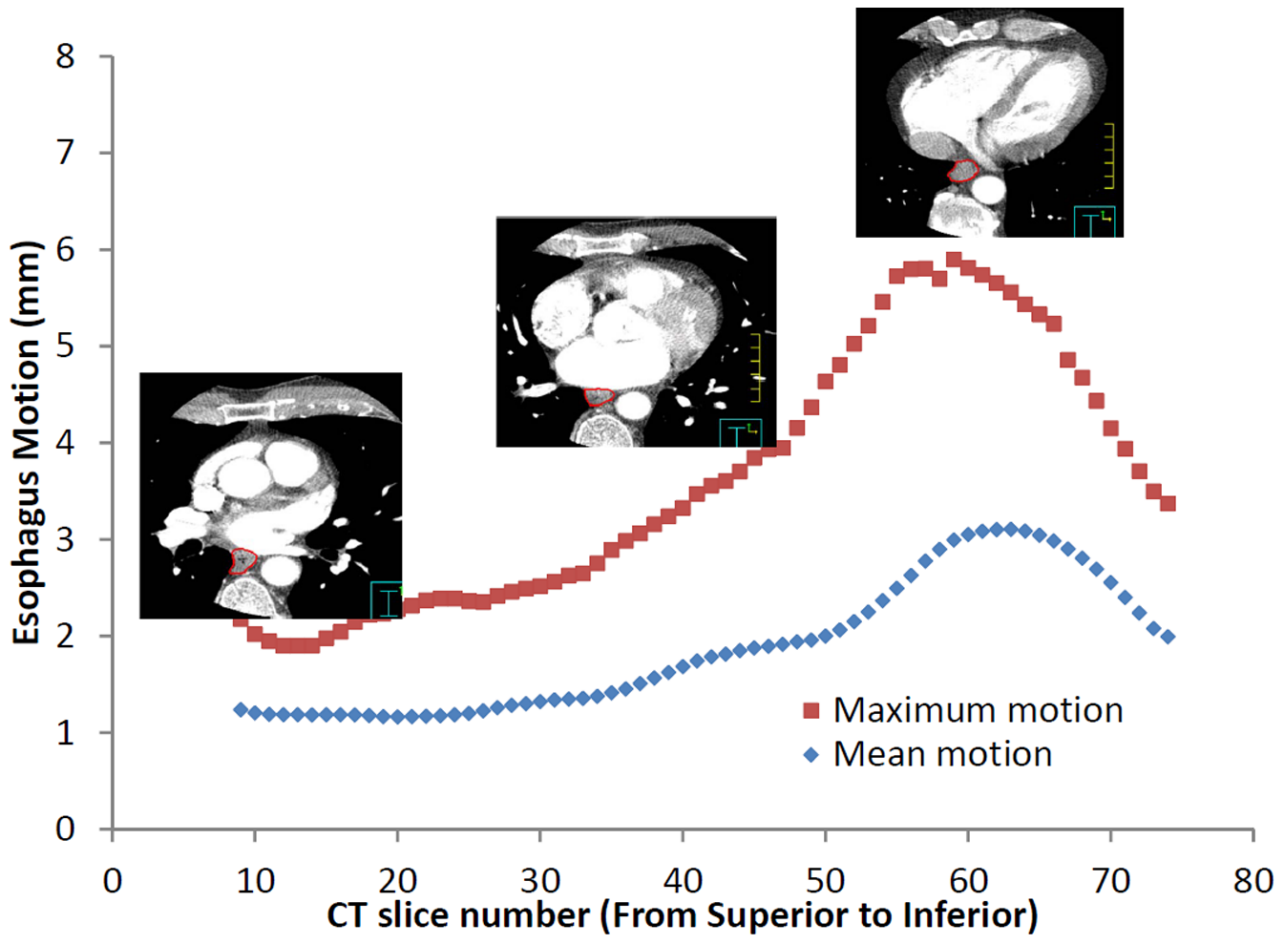
Variation of mean and maximum esophageal motion as a function of CT slice number.

**Figure 3:**
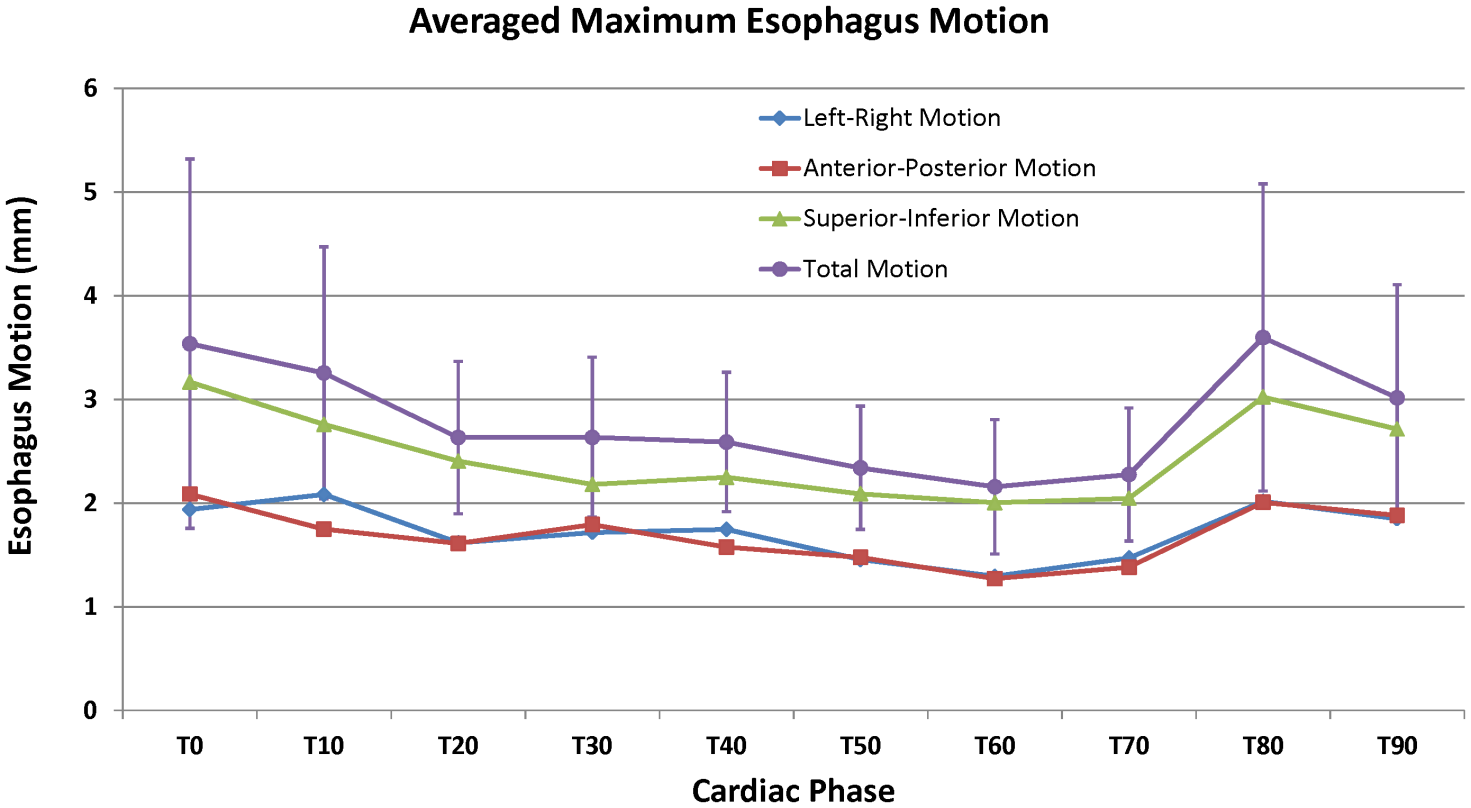
Averaged maximum esophagus motion versus cardiac phase over 26 patients. Error bars for total motion indicate the standard deviation over 26 patients.

The maximum esophageal displacement due to cardiac motion varied between patients, up to 10 mm in the transverse direction. The average maximum esophageal displacement due to cardiac motion for all patients was 5.8 mm (standard deviation: 1.6 mm) in the transverse direction. The extent of motion is patient specific. Table 1 shows the analyzed results for all 26 patients. The mean and maximum peak-to-peak motion of the esophagus was a function of the superior-inferior position, as shown in the example of Figure 2. For 21 of the 26 patients, the largest esophageal motion was found in the inferior region. For the remaining 5 patients, the esophageal peak-to-peak motion was constant (within ∼1 mm) throughout the imaged region. When viewing individual axial slices, the largest esophageal motion was in the esophageal wall closest to the heart or the aorta. Figure 3 showed the averaged maximum esophagus motion versus cardiac phase over 26 patients, including total motion magnitude and motions in LR, AP, and SI directions. The motion was found maximum at T80 with 3.6 mm and minimum at T60 with 2.2 mm. Overall, the motion was larger at phases T0, T10, T80, and T90, where the electrocardiogram R-wave occurs. Figure 3 also showed that SI direction had slightly larger motion than LR and AP directions, but this might not be true because images used for analysis had 1.25 mm resolution in SI dimension but 0.35 mm in LR and AP dimensions, thus resulting the motion calculation in SI direction was less accurate than LR and AP directions.

**Table 1:** The analyzed results for 26 patients.

No.	Mean motion (mm)					Maximum motion (mm)				
	LR	AP	SI	Axial	Total	LR	AP	SI	Axial	Total
1	1.8	2.9	5.0	3.4	6.1	5.3	5.3	12.7	6.0	12.9
2	2.0	1.6	2.0	2.5	3.3	6.3	3.4	6.4	6.7	8.4
3	1.6	2.7	2.9	3.2	3.8	4.0	6.5	8.7	7.0	9.8
4	1.4	1.3	2.1	1.9	2.8	3.1	2.6	5.8	3.8	6.0
5	1.5	1.2	2.1	1.9	2.7	4.6	3.8	4.5	5.9	7.1
6	1.1	1.1	2.5	1.6	2.9	3.0	3.1	7.4	3.7	7.5
7	2.1	2.2	2.5	3.0	3.5	4.8	4.0	6.1	5.8	8.3
8	2.0	1.7	2.4	2.5	3.4	5.8	4.5	4.9	6.3	7.7
9	1.6	1.9	4.0	2.5	4.7	6.2	5.5	8.6	8.2	10.2
10	2.1	1.9	3.5	2.8	4.5	6.5	4.9	10.4	7.4	11.6
11	1.4	1.3	2.1	1.8	2.7	4.6	2.9	6.4	5.3	6.9
12	1.7	1.6	3.4	2.2	4.0	4.1	3.8	8.4	5.0	8.4
13	1.0	1.3	1.8	1.6	2.4	3.2	3.6	5.0	4.8	6.2
14	3.1	2.5	4.9	4.0	6.3	8.8	5.6	9.8	10.0	13.7
15	1.3	1.5	1.9	2.0	2.8	3.8	4.5	5.0	4.8	5.7
16	3.3	5.3	4.3	6.2	7.6	6.0	7.4	7.1	8.7	11.0
17	1.5	1.3	2.4	2.0	3.1	4.3	3.6	8.2	5.1	9.4
18	2.5	2.1	3.9	3.3	5.2	6.6	4.5	11.3	6.9	12.8
19	1.8	1.6	2.1	2.2	3.1	3.3	3.8	4.0	4.0	5.5
20	1.4	1.6	1.8	1.9	2.5	4.7	3.4	3.7	5.5	6.0
21	1.6	1.5	3.7	2.2	4.3	5.1	3.7	7.8	5.4	9.4
22	1.2	1.5	1.9	1.8	2.6	2.6	2.8	4.0	3.3	4.9
23	1.7	1.3	2.4	2.2	3.3	6.4	3.8	4.9	6.6	8.0
24	1.6	1.9	3.1	2.5	4.0	3.6	4.5	9.4	5.5	9.9
25	1.1	1.1	1.6	1.5	2.2	3.0	3.0	4.9	4.0	5.2
26	1.3	1.4	2.9	1.7	3.4	3.3	2.8	6.1	3.8	6.6
Mean	1.7	1.8	2.8	2.5	3.7	4.7	4.1	7.0	5.8	8.4
SD	0.6	0.9	1.0	1.0	1.3	1.5	1.2	2.4	1.6	2.5

For each patient, and for each CT slice, the mean and maximum motions at each direction—left-right (LR), anterior-posterior (AP), and superior-inferior (SI)—and the total motion magnitudes were calculated. Then, for each patient, the maximum values of these mean and maximum motions over all slices were calculated and presented in this table. The mean and standard deviation (SD) were calculated for all 26 patients as well. Because the motion in SI direction was not reliable and difficult to validate, we also reported the motion in the transverse (Axial) direction without taking into account the SI motion.

## Discussion

The results of this study showed that parts of the esophagus move more than the rest of the esophagus due to cardiac motion. To our knowledge, this is the first study to assess the impact of cardiac motion on the position of the esophagus. In fact, only a few studies have assessed the impact of cardiac motion on radiation therapy at any site. Steppenwolde et al. reported that the cardiac beat can cause a displacement of 1–4 mm in the position of markers in the lung, depending on their location, which ranged from 15 mm to 65 mm from the cardiac or aortic wall [1].

The esophagus is subject to other sources of motion, including respiratory motion and intrafraction motion (e.g., relative shifts in position over an interval of several minutes). Pan et al. reported that the esophagus clearly moves during breathing, and the magnitude depends largely on the individual and on the level of the esophagus, ranging from 5 mm in the AP and LR directions at the thoracic inlet to larger than 10 mm at the gastroesophageal junction in these two directions [14]. Several authors have evaluated the respiratory motion of esophageal cancers (rather than healthy esophagus studied here). Yaremko et al. [15] used deformable registration to map the respiratory motion of the gross tumor volume for distal esophageal cancer and found that the mean tumor motion was 9 mm (95^th^ percentile: 20 mm) for a total motion magnitude. Patel et al. conducted a similar study that included several tumors in the proximal two-thirds of the esophagus (corresponding closer to the anatomic region we assessed in our study), and the total displacements due to respiratory motion were less than 4 mm in the transverse direction [16]. Cohen et al. evaluated the intrafraction motion of the esophagus [4]. They compared CT images taken using a CT-on-rails system before and after radiotherapy and found that intrafraction motion of the esophagus in LR and AP directions was greater than 5 mm for 13% of cases and greater than 10 mm for 4% of cases. Thus, for some parts of the esophagus that are in the vicinity of the heart, the impact of cardiac motion (from our study) appears to be comparable to that of respiratory motion (compared with the data of Pan et al). Away from the heart, in the distal region, respiratory motion likely dominates, although our study did not include images away from the heart.

## Conclusion

In conclusion, cardiac motion results in displacements of 5–10 mm for some parts of the esophagus, depending on patient and location. This magnitude is similar to that of other sources of motion, including respiratory motion and intrafraction motion. A larger cardiac motion will result into larger esophagus motion in a cardiac cycle.
